# Higher digital embracement is associated with lower levels of loneliness among late middle-aged and older adults

**DOI:** 10.3389/fpubh.2025.1721185

**Published:** 2026-01-09

**Authors:** Anna K. Dahl Aslan, Kerstin Thelander, Pernilla Bjerkeli, Martin Gellerstedt

**Affiliations:** School of Health Sciences, University of Skövde, Skövde, Sweden

**Keywords:** digital divide, digital health, loneliness, old age, older adults, public health

## Abstract

**Introduction:**

Loneliness, a global health problem, increases with advancing age. The digitalization of society has the potential to either increase or decrease loneliness. This study aims to investigate the cross-sectional association between digital living and loneliness in the context of other risk factors in a sample of late middle-aged and older adults, using a measure of embracement of digitalization in daily life.

**Methods:**

In total, 441 Swedish adults (response rate 44%) aged 55 to 93 years of age who responded from December 2023 to January 2024. Embracement of digitalization was measured using the Digital Living Index, and loneliness with the UCLA Loneliness Scale.

**Results:**

The mean score for perceived loneliness was 35.64 (SD = 10.55), positioning the participants at the threshold between low and moderate levels of loneliness. In the final multivariate linear regression model, including established risk factors for loneliness, low digital living was estimated to be 3.3 and 4.1 units higher in loneliness compared to mid and high digital living (*p* = 0.005), respectively. Mental health was estimated to be the strongest predictor of loneliness, with a difference of 14.1 units between bad or very bad mental health and very good (*p* < 0.001).

**Conclusion:**

Higher digital living appears to be associated with lower levels of loneliness even when other established risk factors for loneliness are controlled for. Supporting late middle-aged and older adults to overcome the digital divide, from access and use to embracement, could potentially be a tool to battle loneliness, and hence to improve public health.

## Introduction

Loneliness is defined as “a subjective unpleasant feeling of lack of or loss of companionship. It happens when there is a mismatch between the quantity and quality of the social relationships that we have, and those that we want” (1). Beyond the individual mental suffering experienced by persons who feel lonely, they are also at increased risk of negative physical and mental health outcomes and decreased longevity, at a magnitude that has been compared to smoking fifteen cigarettes a day (2, 3). This has led the World Health Organization to identify loneliness as a global health threat (4), emphasizing the urgency to develop preventive measures that are directed towards groups in society that are at an increased risk of loneliness, and to identify the underlying reasons for loneliness (5).

It is well-known that one of the groups in society at an increased risk of loneliness are older adults (65 years of age and older) (6). The prevalence of loneliness in old age varies depending on the context, the questions used, and the severity, ranging from 5 to 50% (7). In general, the prevalence of loneliness is higher and more severe among the older old (8), although not consistently (6), and older adults with disabilities and/or living in institutions (9, 10). The roots of loneliness are multifactorial (11). While a great focus in the literature has been on elucidating the individual risk/protective factors for loneliness, such as age, marital status, living conditions, socioeconomic status, fewer studies have focused on the contextual factors (5), also sometimes referred to as structural factors. The contextual factors are not only important in themselves, but also for the potential to affect individual factors. One such contextual factor is digitalization.

It is possible to draw from various theoretical standpoints origin from research on aging and loneliness to explain the association between digitalization and loneliness. The Social Network Theory explains social behavior by focusing on the structure and quality of individuals’ relationships and how these patterns influence well-being (12). In relation to loneliness, the theory suggests that the size, density, and closeness of a person’s social network shape their access to social support and meaningful connection. Individuals with small, sparse, or fragmented networks are more likely to experience social isolation, increasing the risk of loneliness, while rich, cohesive networks can buffer against it. Thus, social network theory links loneliness to characteristics of one’s social ties. However, since this study and many other studies focuses on the subjective feeling of loneliness is motivated to first most use the Cognitive Discrepancy Model as it focus on the *perceived discrepancy* between experienced and desired social relationships (1). The focus is on individual expectations, interpretations, and evaluations, not on objective social conditions. This makes the Cognitive Discrepancy Model inherently subjective.

Access and use of digital devices and internet can increase both the frequency and accessibility of social contact, allowing individuals to maintain relationships despite constraints related to distance, mobility, or health. It can also strengthen the perceived availability of social support and connectedness to the society. Digital environments also offer opportunities to form new connections—both strong and weak ties—through interest-based communities. If these digitally mediated interactions align with individuals’ relational goals and provide meaningful forms of companionship or support, the discrepancy between desired and actual social relationships may diminish and thereby decrease loneliness.

On the other hand, the digitalization may also exacerbate loneliness by widening the gap between desired and actual social relationships. Although digital environments can increase the opportunities for social interactions, the digital environments like social media often also contain idealized images of social connectedness, leading individuals to revise their expectations upward and perceive their own relationships as insufficient. The social expectations can then become difficult to fulfil. Furthermore, digital communication may provide interactions that are frequent, but lack the emotional depth or intimacy individuals desire, increasing the discrepancy between the quality of contact sought and that actually experienced. For some groups—particularly older adults or those with limited digital literacy—the shift to digital communication can reduce access to meaningful interactions as older adults might find them difficult to use. As a result, digitalization may intensify perceptions of unmet relational needs, and hence lead to increased loneliness.

Many of the above-mentioned examples implies that the persons have access to and use internet as well as social media. However, this has historically not been the case for many older adults. Expectedly, this is reflected in the literature, where the vast majority has focused on the first two levels of digital divide (access and use) and how it relates to loneliness as the research is performed in contexts or times when older adults’ access and use of digital devices and internet were lower. Thereof, it remains to be explored how the level of loneliness is affected in cohorts of older adults in highly digitalized societies, where the pressure to embrace the digitalization is larger (13).

High levels of access to, and use of, digital devices and internet are common among older adults in highly digitalized contexts, such as the Nordic countries (14). This is partly because essential services increasingly adopt a “digital only” format, in many cases leaving the individual no choice (13). Among Swedish adults, including those aged 65 to 75 years of age, access and daily internet use is reported to approaching 100%. Although it somewhat lower among the oldest of older adults, access and daily internet use is increasing in this group too (15). Still, this is likely an overestimation (16), and around 50% of Swedish older adult internet users report that they avoid some or many internet services, as they consider themselves to lack confidence, or due to worries and fears (15, 17). In other words, although at a first glance it may appear that older Swedish adults have embraced digitalization, there is heterogeneity regarding to what extent older adults have access, use, and benefit from digitalization. This heterogeneity makes it possible to explore how the embracement of digitalization is related to loneliness in a highly digitalized context.

Recent reviews (18, 19), suggest that the use of digital devices and internet or social media are associated with lower levels of loneliness and higher social participation, although the effect size generally is small (19). It has, for example, been shown that, among middle-aged and older Chinese adults, loneliness decreased when the number of hours of internet use increased, as long as it was not over used (i.e., more than 5 h per day) (20). Further, it has been shown that higher internet use is associated with loneliness over time (21), although there are also claims that there is a lack of robustness in the association (22). Current evidence might be affected by publication biases, promoting studies where significant associations between access and use of digital devices and loneliness are shown, over studies where no significant associations are found. Overall, there is an agreement that there is a lack of studies using a quantitative design based on well-defined samples which use reliable and valid measures as well as considering other established risk factors for loneliness (18, 19, 22).

In addition, as earlier noted, previous research has focused on how access and use of digital devices and internet are related to loneliness. Commonly access and use are assessed by asking what kind of digital devices the participant use and what they use them for, and how often. In the current study, we will extend the current knowledge by use a measure designed to assess to what extent the participants consider themselves to have embraced and benefit from digitalization in their daily life - the Digital Living Index (23). By focusing on whether the respondent find the digitalization useful, the third level of digital divide is assessed. Specifically, the instrument includes questions such as whether the respondents find digitalization useful for administration (banking, tax-declaration), shopping, socialization, learning, or just spending time for fun. In order to compare the results to other findings, a more objective measure of internet use will also be included. Additionally, in line with previous research (21, 24), we will not only include older adults, but also late middle-aged adults (55 to 64 years of age). It is important to not only include the current generations of older adults, but also those that are next in line to enter old age, so as to get a better understanding of the current levels of digitalization and loneliness, which is important for societal planning.

Hence, this study aims to investigate the cross-sectional association between loneliness and digital living in the context of other risk factors for loneliness in late middle-age and old age, using novel measures of embracement of digitalization in everyday life.

## Materials and methods

### Design and data collection

The study was cross-sectional and based on data from the questionnaire “Healthy Ageing in the Digital Society (HeADS)” survey (17), which was approved by the Swedish Ethical Review Authority (reference number 2023–06094-01).

The population was adults aged 55 years or older living in Skövde municipality. A stratified random sampling technique was applied to achieve an equal number of participants and an even gender distribution in the age groups 55–64 years, 65–75 years, and 76 years and above. One thousand participants were invited in December 2023 and 455 responses were gathered by the deadline in the mid of January 2024. Participants could choose to complete the questionnaire either digitally or on paper. Four respondents had answered only a few questions and were therefore excluded from all analyses. Another 10 had not completed the UCLA Loneliness Scale (25) and were therefore excluded from the analyses in the present paper. A total of 441 participants were thus included.

### Dependent variable

Loneliness was measured using the validated UCLA Loneliness Scale, version 3 (25), a 20-item self-report measure designed to assess one’s subjective feelings of loneliness. The scale includes questions like; “How often do you feel like you lack companionship? and “How often do you feel like there is no one you can turn to? The UCLA Loneliness Scale consistently shows high reliability and good validity, with interclass coefficients values of 0.76–0.93 (26). Participants who answered all 20 questions received a score between 20 and 80. Scores above 65 indicate a severe degree of loneliness, scores between 50 and 65 indicate a moderate to high degree, scores between 35 and 50 indicate a moderate degree, and scores below 35 indicate a low degree of loneliness. The internal missing values were few; 427 participants (97%) had responded to all 20 items, the remaining 14 participants (3%) responded to at least half of the items. For missing items, the mean of the existing items was used for imputation.

### Independent variables

The independent variables included digital living, digital usage, gender, age, educational level, living alone or not, place of residence, household economy, occupation, and self-rated physical and mental health.

### Digitalization

The Digital Living Index (23) was used to measure to what extent the participants had embraced digitalization in their daily lives, i.e., if they found digitalization useful for administration (banking, tax-declaration), shopping, socialization, learning, or just spending time for fun. The instrument included 15 items, for instance, “I often use digital technology to acquire new knowledge,” “Shopping online makes my everyday life much easier,” and “Digital technology is an important part of my social life.” This instrument has been face-validated and used in three previous studies with good internal consistency – Cronbach’s alpha: 0.91, 0.90 and 0.93, (23, 27). The alpha coefficient was 0.95 in the present study. The response on each item was ranging from completely disagree to completely agree on a five-degree Likert-scale, numbered 1 to 5. An index was calculated as the average of all items. The index was further categorized into three levels: “Low” (an average lower than 2.5, i.e., mostly disagree), “Mid” (an average between 2.5 and 3.5), and “High” (an average above 3.5, i.e., mostly agree). Those participants who reported that they had neither their own smartphone, tablet, or computer (*n* = 25) were assigned to the lowest category of the Digital Living Index.

As a complement to this subjective measure of digital embracement in daily life, a more objective measure of Digital Usage was also constructed. This was done by summarizing the number of ‘yes’ answers among thirteen items asking about the participants’ use of different digital services during the last 3 months, a method only used to assess digital use. These items included, for example, sending or receiving emails, searching information online, using an app for payment or using social media. This index ranged from 0 to 13, where higher numbers indicate higher usage. Digital Usage was used as a continuous variable.

### Covariates

The covariates were selected based on what has been found to relate to loneliness in other studies (10, 28). This includes age, gender, educational level, living arrangement, place of residence, household economy, occupation and self-rates physical and mental health. All variables were self-reported. As the majority of the sample was community living (17), it was not possible to include institutional living, despite this has been linked to higher level of loneliness (7). Age was categorized into three groups: 55–64, 65–75, and 76 and older. Gender was categorized into male and female, no one responded binary. Educational level was categorized into compulsory school, high school, and higher education. Living arrangement were categorized into living alone or together with someone, either it was a spouse, children, others or a combination of two or more. Place of residence was based on a question if they lived in a city, village or country side. Self-rated household economy was measured by asking the participants to rate “How does your household get by on your present income?” on a five-point Likert-scale ranging from very good to very bad. Bad and very bad were merged into one category in the analysis because the number of responses in these two categories was low. A higher number indicated a better economy. Occupation was categorized into working, retired, or other (including unemployed, being on sick-leave, and studying). Self-rated health was measured using two questions, where one focused on self-rated physical health and the other on self-rated mental health. For both questions, response alternatives were given, on a five-point Likert-scale ranging from very good to very bad. Due to few answers, bad and very bad were merged into one category, for both questions. A higher number indicated better health.

It should also be noted that civil status was known, but since there was a high collinearity between living arrangements and marital status, we needed to choose one of these two. Marital status tells whether someone is legally married, but it does not capture whether they actually share their everyday environment with another person, although it is likely. Living arrangement—whether someone lives alone or with another person—directly measures the presence or absence of daily companionship, which is more proximally related to loneliness. Hence, we choose to proceed with living arrangement.

### Statistical analysis

The analytical process was of an explorative nature. Firstly, the UCLA loneliness score was analyzed with each of the independent variables using bivariate linear regression. The variable occupation was excluded due to its strong multi-collinearity with age. Only 8% were retired in the youngest age category, with 92 and 100% in the mid and high age categories, respectively. The independent variables were also analyzed in relation to the Digital Living Index, using Pearson Chi-square tests.

In the next step, all the independent variables with a bivariate *p*-value below 0.20 were included in a multiple linear model. The 0.2 cut-off was set higher than the overall significance level for the study (*p* < 0.05) to allow for inclusion of variables that might be of importance for the multiple analysis, even if their individual statistical significance was not strong. In the final step, independent variables with a *p*-value above 0.20 in the multivariate model were excluded. This final model was considered the primary result of the study. Due to the explorative nature of these analyses and the many tests performed, significant *p*-values in the final model are regarded as an indication of potential associations rather than conclusive evidence.

In order to further scrutinize the association between digitalization and loneliness, a sensitivity analysis with digital usage was performed. This was done by replacing the Digital Living Index with Digital Usage in the final multivariate linear regression model.

Statistical Packages for Social Science, SPSS, version 28, were used for all data analysis.

## Results

The overall mean score for perceived loneliness among the study participants was 35.64 (SD = 10.55) on the UCLA Loneliness Scale, positioning them at the threshold between low and moderate levels of loneliness.

The mean age was 70 years; the youngest respondent was 55 years of age and the oldest 93. Twenty-five participants (5.7% of the total sample) were 85 years of age and older. Further descriptives of the sample are presented in Table 1.

**Table 1:** Descriptives and bivariate analyses between independent variables and loneliness.

Variable	*n* (%)	Loneliness mean (sd)	*p*-value
Total (*n* = 441)		35.6 (10.6)	
Age (*n* = 440)			
55–64 years	135 (30.7)	33.3 (9.6)	<0.001
65–75 years	163 (37.0)	34.8 (10.13)	
76 years and older	142 (32.3)	38.9 (11.1)	
Gender (*n* = 439)			
Male	216 (49.2)	36.1 (10.67)	>0.20
Female	223 (50.8)	35.2 (10.46)	
Education (*n* = 426)			
Compulsory school	103 (24.2)	39.2 (10.5)	<0.001
High school	194 (45.5)	35.7 (10.9)	
University/college	129 (30.3)	32.3 (8.9)	
Living condition (*n* = 424)			
Living with someone	306 (72.2)	33.7 (9.4)	<0.001
Living alone	117 (27.6)	40.3 (11.2)	
Place of residence (*n* = 432)			
City	330 (76.4)	35.8 (10.6)	>0.20
Village	43 (9.9)	35.5 (11.3)	
Countryside	59 (13.7)	34.5 (9.4)	
Household economy (*n* = 432)			
Very good	151 (34.9)	31.8 (8.7)	<0.001
Good	176 (40.7)	35.8 (9.8)	
Reasonable	77 (17.8)	39.8 (12.9)	
Bad/very bad	28 (6.5)	43.5 (10.1)	
Physical health (*n* = 439)			
Very good	71 (16.2)	30.9 (7.9)	<0.001
Good	189 (43.1)	34.0 (9.2)	
Moderate	129 (29.4)	36.9 (10.7)	
Poor/very poor	50 (11.4)	44.4 (12.3)	
Mental health (*n* = 436)			
Very good	149 (34.2)	30.2 (7.8)	<0.001
Good	195 (44.7)	35.5 (9.2)	
Moderate	75 (17.2)	42.2 (9.6)	
Poor/very poor	17 (3.9)	50.3 (13.9)	
Digital Living Index (*n* = 435)			
High	247 (56.8)	33.4 (9.3)	<0.001
Mid	124 (28.5)	36.9 (9.6)	
Low	64 (14.7)	41.6 (13.9)	

Descriptive statistics presented are means and standard deviations (sd) from the UCLA Loneliness Scale. *p*-values are from bivariate linear regressions.

### Bivariate analyses

Results from the bivariate analyses between the independent variables and loneliness are presented in Table 1. There was no significant association between gender and place of residence with loneliness (*p* > 0.20). Higher age, lower educational level, living alone, lower household economy, lower physical health, lower mental health, and lower digital living were all significantly associated with higher levels of loneliness.

The Digital Living Index was analyzed in relation to the other independent variables; see Table 2. A low degree of digital living was more common among older adults, among those with low educational levels, living alone, lower household economy, and poor physical and mental health. There was no significant difference between men and women in Digital Living Index and likewise related to place of residence.

**Table 2:** Digital living in relation to age, gender, education, living conditions, household economy, and physical and mental health.

Variable	*n*	Digital living			*p*-value ^d^
		Low ^a^	Medium ^b^	High ^c^	
		n (%)	n (%)	n (%)	
Total	435	64 (14.7)	124 (28.5)	247 (56.8)	
Age (*n* = 434)					
55–64 years	135	5 (3.7)	32 (23.7)	98 (72.6)	<0.001
65–75 years	162	13 (8.0)	56 (34.6)	93 (57.4)	
76 years and older	137	45 (32.8)	36 (26.3)	56 (40.9)	
Gender (*n* = 433)					
Male	214	35 (16.4)	60 (28.0)	119 (55.6)	*p* > 0.20
Female	219	28 (12.8)	64 (29.2)	127 (58.0)	
Education (*n* = 430)					
Compulsory school	105	31 (30.7)	38 (37.6)	32 (31.7)	<0.001
High school	196	23 (11.9)	51 (26.4)	119 (61.7)	
University/college	129	7 (5.5)	30 (23.6)	90 (70.9)	
Living condition (*n* = 418)					
Living with someone	304	29 (9.5)	84 (27.6)	191 (62.8)	<0.001
Living alone	114	27 (23.7)	39 (34.2)	48 (42.1)	
Place of residence (*n* = 427)					
City	325	45 (13.8)	91 (28.0)	189 (58.2)	*p* > 0.20
Village	43	8 (18.6)	11 (25.6)	24 (55.8)	
Countryside	59	8 (13.6)	20 (33.9)	31 (52.5)	
Household economy (*n* = 426)					
Very good	150	17 (11.3)	32 (21.3)	101 (67.3)	0.002
Good	173	22 (12.7)	56 (32.4)	95 (54.9)	
Reasonable	75	17 (22.7)	20 (26.7)	38 (50.7)	
Bad/very bad	28	7 (25.0)	13 (46.4)	8 (28.6)	
Physical health (*n* = 433)					
Very good	71	5 (7.0)	12 (16.9)	54 (76.1)	<0.001
Good	186	15 (8.1)	54 (29.0)	117 (62.9)	
Moderate	128	29 (22.7)	43 (33.6)	56 (43.8)	
Poor/very poor	48	15 (31.3)	14 (29.2)	19 (39.6)	
Mental health (*n* = 430)					
Very good	149	15 (10.1)	32 (21.5)	102 (68.5)	0.001
Good	192	25 (13.0)	59 (30.7)	108 (56.3)	
Moderate	72	19 (26.4)	23 (31.9)	30 (41.7)	
Poor/very poor	17	4 (23.5)	7 (41.2)	6 (35.3)	

a Low digital living represents a Digital Living Index ≤2.5.

b Mid digital living represents a Digital Living Index between 2.5 and 3.5.

c High digital living represents a Digital Living Index above 3.5.

d *P*-values are from Pearson Chi-square tests.

Values are numbers and proportions of participants and *p*-values from Pearson Chi-square tests. Digital living was measured using the Digital Living Index.

### Multiple linear models

In the first linear model, independent variables with a *p*-value below 0.20 in the bivariate analyses were included. All independent variables except age and education had a p-value below 0.20; Table 3. Thus, these two independent variables were excluded, while the remaining were used in the final linear model.

**Table 3:** Anova-table for the multiple linear regression model, with loneliness as dependent variable and including all independent variables with a *p*-value below 0.2 from the bivariate analysis (presented in Table 1).

Variable	Sum of squares	df	Mean square	F	*p*-value
Corrected model	13,747.2	16	859.2	11.7	<0.001
Intercept	194,711.2	1	194,711.2	2,657.5	<0.001
Age	148.7	2	74.3	1.0	>0.20
Education	50.0	2	25.0	0.3	>0.20
Living condition	603.0	1	603.0	8.2	0.004
Household economy	465.0	1	603.0	8.2	0.098
Mental health	3,348.0	3	1,116.0	15.2	<0.001
Physical health	526.2	3	175.4	2.4	0.068
DLI	397.7	2	198.9	2.7	0.068
Error	27,622.2	377			
Total	528,240.1	394			
Corrected total	41,369.4	393			

Loneliness was measured using the UCLA Loneliness Scale. R square = 0.33.

In the final model, all independent variables were significant (*p* < 0.05) except physical health (*p* = 0.061); Table 4. The coefficient of determination was unchanged compared to the previous model (R square = 0.33). The parameter estimates in the final model show that digital living was estimated to be 3.3 and 4.1 units higher in loneliness compared to mid and high DLI, respectively. This is similar to the estimates for household economy. The difference between very good economy and bad or very bad economy was 4.4 units. Levels of loneliness for those living alone was estimated as 3.2 units higher compared to not living alone.

**Table 4:** Multivariate linear regression with loneliness as dependent variable.

Parameter	B	SE	*p*-value	95% C.I. for B		Overall *p*-value
				Lower	Upper	
Living alone or not						0.002
Not living alone	−3.2	1.0	0.002	−5.2	−1.2	
Living alone (ref.)	0					
Economy						0.046
Very good	−4.4	2.0	0.026	−8.2	−0.5	
Good	−2.2	1.9	>0.20	−5.9	1.5	
Reasonable	−1.6	2.0	>0.20	−5.6	2.3	
Bad/very bad (ref.)	0					
Mental health						<0.001
Very good	−14.1	2.4	<0.001	−18.8	−9.3	
Good	−10.1	2.3	<0.001	−14.6	−5.5	
Fairly good	−5.7	2.4	0.017	−10.4	−1.0	
Bad/very bad (ref.)	0					
Physical health						0.061
Very good	−4.4	1.9	0.023	−8.1	−0.6	
Good	−4.1	1.6	0.011	−7.2	−0.9	
Fairly good	−3.9	1.5	0.012	−6.9	−0.8	
Bad/very bad (ref.)	0					
Digital living						0.005
High	−4.4	1.4	0.001	−7.1	−1.8	
Mid	−3.3	1.4	0.020	−6.2	−0.5	
Low (ref.)	0					

This is the final model including only independent variables with an *p*-value below 0.2 in the previous model (presented in Table 3). Values are parameter estimates, standard errors, p-values and confidence intervals (95%) for each category of the independent variables as well as the overall p-value for each independent variable. Loneliness was measured using the UCLA Loneliness Scale. R square = 0.33.

Mental health was the independent variable with the highest coefficients. The differences in loneliness scores between bad or very bad mental health compared to very good is estimated as 14.1. It is also noteworthy that the loneliness score successively decreases for every step in better mental health. This pattern cannot be seen in physical health, where loneliness was at the same level for all categories, except when physical health was bad or very bad. Even though physical health wasn’t significant in the overall test, the difference between bad or really bad physical health and all other levels of physical health were significant (*p* < 0.05).

In a complementary analysis, the Digital Living Index was replaced by the measure Digital Usage. This variable was added as a covariate instead of the Digital Living Index in the multiple linear model. The estimates for all other variables remained more or less unchanged, and the estimate for Digital Usage was −0.45 (*p* = 0.024), meaning that loneliness decreased in average by 0.45 units with every extra digital application or service used, assuming all other factors held constant.

On average, the participants in the group with a high score on the Digital Living Index reported using 6.5 more digital services compared to participants in the group with low scores on the Digital Living Index, and 6.5 times 0.45 equals 2.9, slightly lower than the difference of 4.4 found between low and high levels of digital living. This illustrates that the models with the subjective and the objective measure of digitalization in daily life are consistent, both being significant and with estimates of nearly the same magnitude.

## Discussion

This study indicates that higher levels of digital living are associated with lower levels of loneliness among late middle aged and older adults in a highly digitalized society. The association remained significant even when established individual risk factors for loneliness are controlled for, such as living alone and poorer mental health. The magnitude of the association between high digital living and feeling lonely seems to be equal to having a very good self-reported economy and very good self-reported health, and potentially at a higher magnitude than the effect of co-habiting. However, the largest effect is seen for self-reported mental health, where those who consider themselves to have very good mental health have substantially less risk of loneliness compared to those that self-report having bad or very bad mental health. Very good mental health is of a three-fold magnitude compared to all the other risk factors.

### Loneliness, digitization, and the digital divide

Consistent with previous reviews (18, 19), our findings suggest that older adults’ levels of digitalization play a role in loneliness. While previous quantitative research has generally been performed in contexts where the access to and usage of digital devices is still low among older adults, this study extends the finding to a context where almost all older middle-aged and older adults have access to and use digital devices and internet daily.

In relation to the time aspect, it is notable that several of those publications that do not report a significant association between internet use or use of social media and loneliness are studies with older publication dates (24, 29, 30). It is possible, that today, when more persons use digital devices and the internet, those that do not, becomes an even more selected group, with less resources, which also might affect the level of loneliness. For example, persons with no or smaller social networks might have less persons that encourage them to use digital devices and the internet (31). Likewise, they might not see the point of using digital communication if they have no family or friends to interact with or do not want to reveal how small their network is. However, it might also be those digital social interactions per see helps fulfil the persons need for social interaction, and thereby closes the gap between the amount social interactions a person perceive and desire, in line with the cognitive discrepancy theory (1).

The finding that it is not only the use of digital devices and internet that is associated with loneliness, but also that the embracement of digitalization is important, is novel. The latter indicates that it might be important to make sure that older adults move beyond the first two levels of digital divide (access and use) to also reach the third level (benefit).

Another aspect, which provides further evidence for the importance of digital living in relation to loneliness, is that the magnitude of the association with loneliness was at about the same level or higher than all other factors, except self-reported mental health (see below). It is not hard to imagine all the benefits that digitalization might provide for late middle-aged and older adults. For example, to be able to maintain contact with family and friends when there is physical distance. Being able to master their own digital devices, can also contribute to feelings of independence and satisfaction (32, 33), which is related to well-being and quality of life.

A surprising finding was that the magnitude of the association between high digital living and loneliness is *potentially* higher than the association between living alone and loneliness. A plausible explanation is that Sweden is one of the countries in the world with the largest number of one-person households (34), not least among older adults. Swedish older adults living alone might be better prepared to tackle single living, as the norm is to live alone and not together with your children, and accordingly the expectations might differ (35). In fact, several European and international studies show that in regions where cohabiting is more common, e.g., Southern and Eastern Europe, the levels of loneliness among older adults is higher than in Northern and Western parts, where single households are more common (7, 36, 37). It is suggested that higher social participation and lower levels of loneliness are enabled in welfare states and high income countries (36). Hence, the finding that digital living is a stronger predictor of loneliness than living alone might be context dependent, and would need to be replicated in other samples.

Although digital living is used as an independent variable and loneliness as the dependent variable in the current study, it is not possible draw any conclusions about the causality due to the study’s cross-sectional design. However, there is a study based on the Health and Retirement Study, which shows that higher internet use is associated with decreased loneliness over an eight-year period (21), lending support for the idea that use of digital devices and the internet might be the driving factor. It should also be mentioned that is also possible that the association between higher digital use and living and less loneliness might be due to some other underlying factor not captured in the current study. Although this might be the case, this is less likely in the current study, as the most established risk factors for loneliness are included in the multivariate model.

Taken together, there are good reasons to believe that digital access, use, and benefit/embracement into daily life have the potential to reduce loneliness among late middle-aged and older adults in a non-invasive manner, although this should not be seen as the only solution. Especially, there is need for initiatives to improve mental health. Still, given the high prevalence and severity of loneliness, the benefits of using digitalization to tackle such loneliness might have a substantial impact on the population level (19). Hence, it is important to start, continue, or intensify interventions, training, and other actions which support older adults’ access, use, and benefit of digital devices and internet. This might also have the benefit to support digital equity and narrowing the digital divide. Not surprisingly, a main incentive for internet use among older adults is to maintain contact with family and friends (38).

### Mental health and loneliness

Given that self-reported mental health was the outstanding strongest predictor of loneliness, with significant increase in loneliness for each increase in worse self-reported mental health, this result needs to be discussed, although it was not the primary interest of the study. The association between mental health and loneliness is particularly relevant in light of Cacioppo’s theory, which posits that long-term loneliness can lead to a negative spiral of self-perception and diminished social confidence (39). This has important implications for healthcare and social services, as it highlights the need to address mental health in efforts to reduce loneliness, and, conversely, to reduce loneliness as a strategy to prevent mental health problems. This is in line with an early study on the association between use of social media, loneliness, and physical and mental health, were loneliness mediate the association between social media use and physical and mental health (40). Greater awareness of these connections could encourage more professionals to proactively discuss mental health and loneliness with older adults and to implement targeted digital interventions.

In conjunction with the current study’s findings on digitalization and loneliness, these results also raise questions about whether digital social interaction could serve as a less intimidating first step in breaking loneliness, particularly for individuals who struggle with in-person interactions. This would be interesting to explore in future studies.

A previous study including middle-aged and older adults did show a moderating effect of health status, where adults with poor health benefited more from mobile internet use than adults with good health, although the latter also benefited (41). In the current study, only adults with very bad self-reported health felt lonelier, meaning that between those with very good health and bad health, there was no difference in level of loneliness. Given the diverse causes of both loneliness and degree of digitalization, the effectiveness of online social interactions is likely to vary between individuals. Nevertheless, the observed association between mental health and loneliness is noteworthy and underscores the importance of battling both loneliness and mental health.

### Other covariates

Notably, age did not significantly predict loneliness. This finding aligns with another Swedish study on older adults, which suggests that loneliness is not directly caused by age itself, but rather by the social and health challenges associated with ageing, such as changed living and health conditions (42). In other words, as transpires from the current study, it does not matter if you are 55 or 75 years of age if you have the exact same circumstances (the covariates in the current study), but if your level of digital living differs it will likely influence your level of loneliness. This means that even those in late middle-aged might benefit from using and embracing digitalization to counteract loneliness. Hence, interventions and services which support achievement of digital use, skills, and benefits should not only be based on age, which is sometimes the case, in for example the service Digital Coach, which some Swedish municipalities offer to older adults (32).

Regarding gender, previous research on this topic has yielded mixed results (28, 43). Since previous research is not conclusive, our non-significant finding does not seem to stand out. As a matter of fact, in a review of risk factors of loneliness, gender rarely seem to associated with loneliness in multivariate analyses (28). As there are previous studies based on Swedish sample that indicate that there is no gender difference in loneliness in old age (44) the non-significant gender difference in our study seems reasonable. Economic factors remained significant in the final model, as anticipated. The relationship between financial hardship and loneliness is well established, with self-assessed financial strain (45) and objectively measured income (46) linked to reduced social support, smaller social networks, and higher levels of loneliness. The finding that educational attainment was not a significant factor also aligns with prior research (28). For example, it has been shown that among three key socioeconomic indicators, educational level, income, and occupational status, it was income and occupational position that were significantly associated with loneliness (47). Similarly, another study on access to social support demonstrated that income level had a more significant impact than education (45). Low income can also restrict participation in social activities, as many such opportunities involve financial costs.

### Limitations and strengths

Key strengths of our study are the rigid data collection using an analytical method that incorporates multiple known risk factors for loneliness, measured with valid and reliable scales within the same model, allowing for a direct comparison of their impact on loneliness. To assess loneliness, being the primary outcome measure of this study, we utilized the validated UCLA Loneliness Scale (25). This instrument has demonstrated high validity and reliability across various age groups and populations, including young students and older adults (26). However, a validated Swedish translation is currently unavailable, which could be a study limitation. Given the current sample, including mainly community living older adults without home help service (17), the level of loneliness, positioning them at the threshold between low and moderate levels of loneliness, seem reasonable.

The measuring instrument “Digital Living” has been used in previous studies with high internal consistency (23). We selected the Digital Living Index since it reaches beyond previously used measures that focus on access and use of digital devices and the internet. The complementary analysis, in which the variable Digital Use is consistent with DLI, increases the study’s validity. However, a limitation of this study is that we were unable to distinguish between different types of engagement with digital devices and the internet, including that quality of the interaction, that could have distinct implications for the outcomes examined. This needs to be addressed in future studies.

As for all studies on ageing, there is a selection bias for healthier older adults. Hence the results cannot be generalized to all older adults. In particular, those with higher health care needs, living in nursing homes, and being of a higher age did not respond to the current questionnaire (17). It is also likely that older adults who are not interested in digitalization are less likely to respond to a questionnaire entitled “Healthy Ageing in the Digital Society” (17), likely leading to an overestimation of older adults’ access, use, and benefit of digital devices and internet (16).

Although the respondents in the study likely were more digitally engaged than the general population, this does not necessarily mean that the relationship between digitalization and loneliness was affected. An exploratory analysis comparing the final model between those who responded immediately and those who responded after a reminder showed consistent results. The same pattern also emerged when comparing respondents who replied by regular mail with those who responded digitally. These analyses indicate that the relationship between digitalization and loneliness likely is robust.

## Conclusion

This study indicates that digital living might be related to loneliness and that it could be a factor of greater magnitude than living alone. Hence, to combat loneliness it is important to not only support older adults’ achievement of digital access and use, but also to provide support to the level whereby embracement and benefit of digital devices and the internet is achieved. This support should not be limited to adults of a certain age, but to those who have a need, as age per se is not related to loneliness. To combat loneliness, it is also of utter importance to promote mental health, as low mental health has been found to be the strongest predictor of higher levels of loneliness.

## Data Availability

The raw data supporting the conclusions of this article will be made available by the authors, without undue reservation.

## Author’s note

This research was undertaken as part of Kerstin Thelander’s master’s thesis at the School of Health Sciences, University of Skövde., Hogskolan i Skovde Institutionen for halsovetenskaper, Skövde, Sweden.
